# Avaliaáo do uso de álbum seriado sobre amamentaáo como estratégia de intervengo educativa no puerpério*

**DOI:** 10.15649/cuidarte.2880

**Published:** 2023-09-09

**Authors:** Iara Angélica da Silva Lima, Paula Gabrielle Gomes Candido, Romila Martins de Moura Stabnow Santos, Laise Sousa Siqueira, Leonardo Hunaldo dos Santos, Marcelino Santos, Floriacy Stabnow Santos

**Affiliations:** 1 Universidade Federal do Maranháo, Imperatriz, Maranháo, Brasil. Email: iaraangelica@live.com Universidade Federal do Maranhão Universidade Federal do Maranháo Imperatriz Maranháo Brazil iaraangelica@live.com; 2 Secretaria Municipal de Saúde de Marabá, Marabá, Pará, Brasil. E-mail: paula.candido@discente.ufma.br Secretaria Municipal de Saúde de Marabá Secretaria Municipal de Saúde de Marabá Marabá Pará Brazil paula.candido@discente.ufma.br; 3 Universidade Federal do Maranháo, Imperatriz, Maranháo, Brasil. Email: romila.martins@discente.ufma.br Universidade Federal do Maranhão Universidade Federal do Maranháo Imperatriz Maranháo Brazil romila.martins@discente.ufma.br; 4 Hospital Municipal de Imperatriz, Imperatriz, Maranháo, Brasil. E-mail: ls.siqueira@ufma.br Hospital Municipal de Imperatriz Imperatriz Maranháo Brasil ls.siqueira@ufma.br; 5 Universidade Federal do Maranháo, Imperatriz, Maranháo, Brasil. E-mail: leonardo.hunaldo@ufma.br Universidade Federal do Maranhão Universidade Federal do Maranháo Imperatriz Maranháo Brazil leonardo.hunaldo@ufma.br; 6 Universidade Federal do Maranháo, Imperatriz, Maranháo, Brasil. E-mail: marcelino.santos@ufma.br Universidade Federal do Maranhão Universidade Federal do Maranháo Imperatriz Maranháo Brazil marcelino.santos@ufma.br; 7 Universidade Federal do Maranháo, Imperatriz, Maranháo, Brasil. E-mail: floriacy.stabnow@ufma.br Universidade Federal do Maranhão Universidade Federal do Maranháo Imperatriz Maranháo Brazil floriacy.stabnow@ufma.br

**Keywords:** Aleitamento materno, Educado em Saúde, Autoeficácia, Breastfeeding, Health Education, Self-efficacy, Lactancia materna, Educación para la salud, Autoeficacia

## Abstract

**Introdujo:**

A amamentaáo é um ato que vai além de nutrir a crianza. A implementaáo de tecnologias educacionais pode favorecer a promoáo do aleitamento materno.

**Objetivo:**

Avaliar o uso de álbum seriado sobre amamentaáo com estratégia de intervengo educativa no puerpério imediato entre puérperas de maternidade pública de acordo com o perfil sociodemográfico e obstétrico.

**Materiais e métodos:**

Estudo quase- experimental de modelo pré e pós-teste com único grupo, realizado entre novembro de 2019 e maro de 2020. Dados coletados através da escala de autoeficácia em aleitamento materno Breastfeeding Self-Efficacy Scale - short form. Avaliou-se a quantidade de mulheres em alta autoeficácia antes e depois da intervengo. Por náo apresentarem normalidade e/ou homogeneidade de variancia, foram realizados testes náo paramétricos de Kruskal-Wallis com post-hoc de Nemenyi.

**Resultados:**

Os resultados demonstraram que houve aumento da autoeficácia após a intervengo, sobretudo em puérperas menores de 18 anos, com Ensino Fundamental, donas de casa, com menos de seis consultas, as orientadas sobre amamentaáo no pré-natal e as que náo amamentaram anteriormente.

**Discussáo:**

O perfil sociodemográfico das genitoras pode representar influencia sobre a autoeficácia na amamentaáo.

**Conclusáo:**

a tecnologia educativa foi eficaz no aumento da autoeficácia materna em amamentar no puerpério imediato.

## Abstract (en)

**Introduction:**

Breastfeeding is an act that goes beyond nourishing the child. The implementation of educational technologies can favor the promotion of breastfeeding.

**Objective:**

To evaluate the use of a flipchart about breastfeeding as an educational intervention strategy in the immediate postpartum period among mothers in a public maternity hospital according to their sociodemographic and obstetric profile.

**Materials and Methods:**

Quasi-experimental study of a pre- and post-test model with a single group, carried out between November 2019 and March 2020. Data collected through the Breastfeeding Self-Efficacy Scale - short form. The number of women with high self efficacy before and after the intervention was evaluated. As they did not show normality and/ or homogeneity of variance, non-parametric Kruskal-Wallis tests with post-hoc Nemenyi tests were performed.

**Results:**

The results showed that there was an increase in self-efficacy after the intervention, especially in puerperal women under 18 years of age, with Elementary Education, housewives, with less than six consultations, those oriented about breastfeeding during prenatal care and those who had not previously breastfed.

**Discussion:**

The sociodemographic profile of mothers may influence self-efficacy in breastfeeding.

**Conclusion:**

educational technology was effective in increasing maternal self-efficacy in breastfeeding in the immediate puerperium.

## Resumen

**Introducción:**

La lactancia materna es un acto que va más allá de nutrir al niño. La implementación de tecnologías educativas puede favorecer la promoción de la lactancia materna.

**Objetivo:**

Evaluar el uso de un rotafolio sobre lactancia materna como estrategia de intervención educativa en el puerperio inmediato en puérperas de una maternidad pública según su perfil sociodemográfico y obstétrico.

**Materiales y Métodos:**

Estudio cuasiexperimental de un modelo de pre y post test con un solo grupo, realizado entre noviembre de 2019 y marzo de 2020. Datos recolectados a través de la Escala de Autoeficacia en Lactancia Materna - forma corta. Se evaluó el número de mujeres con alta autoeficacia antes y después de la intervención. Debido a que no mostraron normalidad y/u homogeneidad de varianza, se realizaron pruebas no paramétricas de Kruskal-Wallis con pruebas post-hoc de Nemenyi.

**Resultados:**

Los resultados mostraron que hubo un aumento en la autoeficacia después de la intervención, especialmente en las puérperas menores de 18 años, con Educación Primaria, amas de casa, con menos de seis consultas, las orientadas sobre lactancia materna durante el prenatal y las que tenían no amamantado previamente.

**Discusión:**

El perfil sociodemográfico de las madres puede influir en la autoeficacia en la lactancia.

**Conclusión:**

la tecnología educativa fue efectiva para aumentar la autoeficacia materna en la lactancia materna en el puerperio inmediato.

## Introdujo

Amamentar é uma agáo que ultrapassa a nutrigáo da crianza e, abrange também o relacionamento entre máe e filho com impactos no estado nutricional da crianza, na capacidade de defesa contra infecgóes, no crescimento e capacidade de aprendizagem e adaptar-se ao ambiente social, além disso, favorece o bem-estar físico e psíquico da máe^1^.

Embora existam evidencias científicas que apontem a excelencia do leite materno, e instituigóes nacionais e internacionais defendam a superioridade da prática do aleitamento materno, a amamentagáo no Brasil, em especial do aleitamento materno exclusivo (AME), está abaixo das taxas preconizadas, e acredita-se que o profissional de saúde possui relevancia na reversáo desse quadro^2^.

Visando um aumento dessas taxas, iniciativas de promogáo, protegáo, apoio e incentivo ao aleitamento materno tem sido desenvolvidas no Brasil, como a Iniciativa Hospital Amigo da Crianza (IHAC), a normatizagáo do Alojamento conjunto (ALCON), a aprovagáo da Norma Brasileira de Comercializado de Alimentos para Lactentes e Crianzas na Primeira Infancia, Bicos, Chupetas e Mamadeiras (NBCAL), o Método Canguru, a Rede Brasileira de Bancos de Leite Humano (Rede BLH)^3^.

Ainda vale ressaltar o Estudo Nacional de Alimentado e Nutrido Infantil - ENANI-201 9^4^ que mostrou as taxas de AME entre crianzas com até seis meses que alcangou a prevalencia de 45,7, sendo que na regiáo Sul os índices alcangaram 53,1% e no Nordeste 38,0%. Posto isto, supóe-se que, possivelmente, as condigóes socioeconómicas podem estar associadas a prática da amamentagáo^5^.

De acordo com uma pesquisa realizada no interior do estado do Maranháo com 174 criangas menores de 12 meses, pode-se identificar que a taxa do AME em criangas menores de seis meses foi de 20,1%^6^. Diversos fatores como o nível de escolaridade da máe, trabalho materno fora do lar, renda familiar dentre outros, influenciam a máe a introduzir alimentagáo complementar o que acaba induzindo ao desmame precoce^7^.

Dessa forma, se faz necessária uma análise para verificar se tais fatores podem contribuir para o desmame precoce, e se esses estáo relacionados a falta de orientagáo das máes durante o período de gestagáo, parto e pós-parto. A fim de que a mulher se sinta segura no seu papel de máe e nutriz, precisa sentir-se devidamente percebida e acompanhada nas suas dúvidas e dificuldades. Compete aos profissionais de saúde informá-la por meio da educagáo em saúde, promovendo o AME até seis meses e a introdugáo de uma alimentagáo complementar adequada após esse período^6^.

Assim, há uma necessidade de esclarecimentos sobre o processo de lactagáo, mostrando os riscos da alimentagáo artificial e promovendo o AME nos primeiros seis meses. Essas orientagóes se iniciam desde as consultas de pré-natal, pasando pelo momento do parto, quando a mulher está no hospital, e váo até o puerpério. No entanto, o objetivo da orientagáo náo é impor a amamentagáo, mas tem como papel primordial informar as máes sobre as vantagens da realizagáo deste ato^8^.

Além disso, a implementagáo de tecnologias educativas pode favorecer mudangas comportamentais e o uso do álbum seriado como tecnologia educativa leve é uma estratégia eficaz para a promogáo da saúde de mulheres, potencializando a educagáo em saúde e aproximando o público alvo do tema trabalhado possibilitando uma assimilagáo real do conhecimento^6^.

Nesse contexto, esta pesquisa objetivou avaliar o uso de álbum seriado sobre amamentagáo com estratégia de intervengáo educativa no puerpério imediato entre puérperas de maternidade pública de acordo com o perfil sociodemográfico e obstétrico.

## Materiais e Métodos

Tratou-se de um estudo quase-experimental de modelo pré e pós-teste com único grupo^9^, com uma abordagem quantitativa, desenvolvido entre novembro de 2019 e margo de 2020, em maternidade pública de referencia regional, no sudoeste do Maranhao.

Foram incluídas no estudo puérperas com neonatos a termo, em alojamento conjunto, com no mínimo seis horas após o parto, que residiam em Imperatriz (MA). Foram excluídas aquelas com problemas cognitivos que pudesse afetar a comunicagao com os pesquisadores, as que possuíam alguma doenga que contraindicasse a amamentagao, mulheres que apresentassem intercorrencias clínicas ou obstétricas e mulheres analfabetas.

Em média, mensalmente, sao realizados cerca de 600 procedimentos entre partos normais e cirúrgicos na maternidade da presente pesquisa. A amostra, considerando o intervalo de confianga de 95%, erro amostral de 5%, obteve-se o quantitativo de 300 puérperas.

A amostra foi determinada por conveniencia e a pesquisa aconteceu em tres etapas: a primeira etapa ocorreu no próprio leito da puérpera, sendo realizada uma entrevista que compreendeu a aplicagao de um pré-teste para avaliar o conhecimento da puérpera sobre o aleitamento materno utilizando- se a escala de autoeficácia em aleitamento materno Breastfeeding Self-Efficacy Scale - short form (BSES-SF)^10^ e coleta de dados sociodemográficos e obstétricos (idade, estado civil, escolaridade, renda familiar, se tem trabalho formal, quantidade de filhos, realizagao de pré-natal, número de consultas, se foi orientada sobre amamentagao no pré-natal, se amamentou anteriormente e dificuldades para amamentar) utilizando um questionário criado pelos pesquisadores.

Na segunda etapa realizou-se uma intervengao educativa com o uso do álbum seriado “Eu posso amamentar o meu filho”, nas enfermarias, uma única vez, com duragao aproximada de 15 a 30 minutos, de forma coletiva, sendo a única intervengao educativa sobre amamentagao realizada entre as puérperas no período de internagao. O álbum é um instrumento criado por uma enfermeira a partir da reflexao dos itens da BSES-SF, dos pressupostos da Teoria de Autoeficácia^10^, é composto por oito telas com ilustragóes com personagens que mostram a realidade vivenciada pela nutriz, sendo que o verso de cada tela é voltado para o profissional.

A terceira etapa foi realizada no mínimo 24 horas após a intervengao educativa com álbum seriado, sendo realizada uma segunda entrevista, quando foi feito o pós-teste aplicando-se novamente a BSES-SF. A BSES-SF foi desenvolvida no Canadá traduzida no Brasil e validada com alfa de Cronbach (=0,74) e contém 14 itens apresentados em dois domínios (Técnico, oito itens; e Pensamentos Intrapessoais, seis itens), sendo que as respostas tem cinco categorias (discordo totalmente; discordo; as vezes concordo; concordo; concordo totalmente), denominada escala de Likert, podendo variar de 14 a 70 pontos^10^. Considerando 14 a 32 baixa eficácia, 33 a 52 média eficácia e 53 a 70 alta eficácia em amamentagao de modo que quanto mais escores obtenha no somatório dos itens, maior a auto eficácia para amamentar^11^. A BSES-SF, através das somas dos escores obtidos consegue identificar se a mulher apresenta comportamento favorável a manter o aleitamento materno, o que indica um maior escore de autoeficácia e reforga a ideia de qua a mulher tem maior motivagao para a prática da amamentagao^11^.

Avaliou-se a quantidade de mulheres em alta autoeficácia (antes e depois da intervengao) aplicando o Teste de McNemar, como descrito em Fontelles^12^. Em seguida, comparou-se a diferenga (Antes - Depois) na Escala de autoeficácia de acordo com as características sociodemográficas e de aleitamento materno. Por nao apresentarem normalidade (Shapiro-Wilk) e/ou homogeneidade de variancia (Levene), foram realizados testes nao paramétricos de Kruskal-Wallis com post-hoc de Nemenyi para comparapóes múltiplas e U Wilcoxon-Mann-Whitney^13^.

Os dados quantitativos foram analisados considerando as frequéncias absolutas e relativas das variáveis categóricas e organizados em tabelas e apresentados de forma descritiva. As informares validadas foram exportadas para o pacote estatístico Stata/MP versao 14.0. Para processamento de dados. O banco de dados foi armazenado no Mendeley Data^14^. Todas as análises foram realizadas no programa IBM SPSS^15^ a 5% de significancia.

A presente pesquisa atendeu aos preceitos éticos e teve aprovapáo do Comité de Ética em Pesquisa da Universidade Federal do Maranhao com o número 3.450.563 e as participantes assinaram o Termo de Consentimento Livre e Esclarecido ou o Termo de Assentimento Livre e Esclarecido no caso das participantes menores.

## Resultados

No que diz respeito a caracterizado do perfil socioeconómico, demográfico e obstétrico das 300 puérperas estudadas, a idade variou de 13 a 40 anos sendo a maioria adultas jovens (262: 87,33%); era casada ou em uniao estável, 61,66% (185); tinham de nove a 12 anos de estudo 73,00% (219); com renda familiar entre 1 e 2 salários mínimos 68,00% (204); eram donas de casa 76,67% (230); tinham 2 filhos 42,00% (126). Fizeram pré-natal 99,33% (298); fizeram seis ou mais consultas 66,00% (198); nao foram orientadas sobre aleitamento materno nas consultas de pré-natal 50,00% (150); 76,00% (228) tiveram experiencia anterior com amamentapáo; praticaram AME até seis meses 24,00% (72) (Tabela 1).

**Tabela 1:** Características sociodemográficas, obstétricas e de amamentaáo de puérperas em maternidade pública, Imperatriz - MA, 2020

Características	n	%
Idade (anos)		
< 18	20	6,66
18 a 30 anos	262	87,33
31 a 40 anos	18	6,01
Estado Civil		
Casada/Uniao estável	185	61,66
Solteira	113	37,66
Divorciada/separada	2	0,68
Escolaridade (anos de estudo)		
Até 9 anos	60	20,00
9 a 12 anos	219	73,00
>12 anos	21	7,00
Renda familiar (salário mínimo*)		
< 1 salário mínimo	90	30,00
1 a 2 salários mínimos	204	68,00
>3 salários mínimos	6	2,00
Situado de trabalho		
Emprego formal	70	23,33
Dona de casa	230	76,67
Quantidade de filos		
1 filho	72	24,00
2 filhos	126	42,00
3 a 5 filhos	99	33,00
> 6 filhos	3	1,00
Fez pré-natal		
Sim	298	99,33
Nao	2	0,67
Número consultas		
< 6	100	33,33
6 ou mais	198	66,00
Nenhuma	2	0,67
Foram orientadas sobre aleitamento		
materno no pré-natal		
Sim	150	50,00
Nao	150	50,00
Experiencia anterior com amamen-		
taao		
Sim	228	76,00
Nao	72	24,00
Tempo de AME**		
<1 mes	17	5,66
2 a 3 meses	70	23,33
4 a 5 meses	68	22,67
6 meses	73	24,34
Nao amamentou	72	24,00
Total	300	100

Fonte: dados da pesquisa *Salário mínimo vigente R$ 998,00. **AME Aleitamento materno exclusivo

Observou- se que após a intervengo educativa, as médias dos escores da BSES-SF aumentaram, nas seguintes variáveis sociodemográficas e obstétricas: puérperas com idade menor de 18 anos (7,41); que tinham até nove anos de estudo (6,73); donas de casa (5,92); as que realizaram seis ou mais consultas (5,92); puérperas que foram orientadas sobre amamentagáo no pré-natal (5,91) e as que nao amamentaram anteriormente (7,00) (Tabela 2).

**Tabela 2:** Caracterizado e comparado entre as médias dos escores da BSES-SF antes e depois da intervendo educativa, de acordo com variáveis sociodemográficas e obstétricas das puérperas, maternidade pública, Imperatriz -MA, 2020

Variáveis	(Depois - Antes)		
	Média	DP	p-valor
Idade			0,009*
< 18 anos	7,41a	4,22	
18 a 30 anos	5,30b	4,40	
31 a 40 anos	3,95b	4,08	
Escolaridade (anos de estudo)			0,002*
Até 9 anos	6,73a	5,11	
9 a 12 anos	5,22a	4,13	
>12 anos	3,05b	3,99	
Situaao laboral			<0,001**
Emprego formal	3,53	3,65	
Dona de casa	5,92	4,46	
Número de consultas pré-natal			0,04**
< 6	5,85	3,96	
6 ou mais	5,92	4,59	
Foram orientadas sobre			0,03**
amamentaao no pré-natal			
Sim	5,91	4,70	
Nao	4,78	4,00	
Já amamentou antes			<0,001**
Sim	4,86	4,03	
Nao	7,00	5,14	

Fonte: dados da pesquisa DP - Desvio-padráo. *Categorias com letras diferentes possuem distribuifáo estatisticamente diferentes pelo teste de Kruskal-Wallis (comparagáo múltipla de Nemenyi) a 5%. **U Wilcoxon-Mann-Whitney. Fonte: Autoríaprópria (2020).

Na Tabela tres é realizado um comparativo da auto eficácia no pré e pós-teste onde a maioria das puérperas obteve alta autoeficácia em amamentar, apresentando 64,00%(192) puérperas no pré- teste e 96,30%(289) no pós-teste. Houve um aumento significativo de 97 puérperas (p<0,001) depois da intervengao em alta autoeficácia. Nenhuma mulher apresentou baixa eficácia nos dois momentos.

**Tabela 3:** Caracterizado e comparado dos escores de autoeficácia em amamentaáo no pré- teste e pós-teste entre puérperas internadas em maternidade pública, Imperatriz - MA, 2020

Alta Autoeficácia				
GERAL	Categoria	n	%	P*
	Antes	192	64,00	<0,001
	Depois	289	96,33	

Fonte: Dados da pesquisa. *Valores de p<0,05, o número de acertos antes e depois diferem significativamente de acordo com o Teste de McNemar a 5% de significancia.

**Figura 1:**
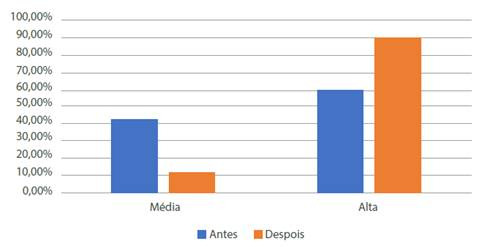
Caracterizado e comparado dos escores de autoeficácia em amamentaáo no pré- teste e pós-teste entre puérperas internadas em maternidade pública, Imperatriz - MA, 2020 Fonte: Dados da pesquisa (2020)

A Figura 1 mostra a comparado dos escores de autoeficácia antes e depois da intrvengáo. Antes da intervengo 41,66%(125) apresentaram média autoeficácia e 58,34%(175) alta autoeficácia e náo houve nenhuma puérpera que tivesse baixa autoeficácia. Após a intervengo, 9,33%(28) apresentaram média autoeficácia e 90,67%(272) alta autoeficácia e novamente nenhuma participante teve baixa autoeficácia.

## Discussao

Destaca-se inicialmente que a idade materna e o grau de instrugáo podem influenciar na decisáo da máe em amamentar ou náo e, estudos indicam que quanto menor for a idade materna e grau de instrugáo, mais dificuldades sáo evidenciadas no processo de cuidado e aleitamento materno^8^^,^^16^. Entretanto, nesta investigado, denotou-se que a intervendo educativa utilizada teve influencia sobre as variáveis de idade menor de 18 anos, e 9 a 12 anos de estudos, aumentando a autoeficácia para amamentar.

Quanto a autoeficácia em amamentar, a maioria das entrevistas apresentou alta autoeficácia, conforme estudos desenvolvidos no interior Minas Gerais que também se aplicou a versáo reduzida da BSES (BSES - Short Form)^17^ e em uma maternidade pública situada no município de Ribeiráo Preto (SP)^18^.

Ao analisar o antes e depois da intervendo educativa, em relado as variáveis de escolaridade é perceptível que ter de 9 a 12 anos de estudo, náo influenciou no fato de a mulher ter alta autoeficácia em amamentar. Em contrapartida aos achados dessa investigado, outros estudos afirmam que mulheres com maior escolaridade, por possuir mais instrugáo, detem mais conhecimento sobre as vantagens do aleitamento materno, levando-as a amamentar por mais tempo. Já as mulheres com menor grau de escolaridade tendem a desmamar precocemente seus filhos, visto que mulheres com pouca instrugáo desconhecem a importancia do aleitamento materno, tornando assim indispensável o uso de intervengóes nesses grupos^19^^), (^^7^.

Nesta investigagáo, a renda familiar da maioria entrevistadas era de um a dois salários mínimos. Estudo realizado em Belém, capital do estado do Pará, mostrou que a maioria das participantes também possuía renda familiar similar^19^. As desigualdades sociais, em especial o nivel socioeconómico, reproduzem-se nas condigóes de saúde afetando positivamente a interrupgáo do AME^20^.

No que se refere a situagáo de trabalho, 76,0% das participantes eram donas de casa. O fato da mulher permanecer em casa por um tempo maior após o nascimento da crianga, pode ser um fator positivo que facilitaria a amamentagáo, considerando que máes que trabalham fora de casa apresentam mais chances de iniciar o desmame no retorno ao trabalho^21^. No presente estudo, a variável ser dona de casa apresentou escore estatisticamente significante para alta autoeficácia após intervengáo educativa, portanto, as participantes apresentaram situagáo favorável para o aleitamento materno.

Quanto a situagáo obstétrica, observou-se que 42,7% das mulheres eram secundigestas e a maioria (76,7%) das mulheres amamentou anteriormente. O fato de a máe ter vivido uma experiencia anterior de amamentagáo pode relacionar-se a efetivagáo do aleitamento materno, pois, a primeira gravidez é um desafio a adaptagáo da mulher como pessoa, um desafio a sua maturidade, a constituigáo da sua personalidade^22^.

Neste estudo as mulheres que náo amamentaram anteriormente apresentaram escore elevado antes e após a intervengáo. Entretanto, as primíparas podem estar mais propensas a interromper o AME devido a sua falta de experiencia anterior, o que pode leva-la a introduzir precocemente alimentos complementares, acreditando que seria necessária a oferta de outro leite^23^^), (^^24^.

Foi possível identificar que a maioria absoluta (99,7%) das puérperas realizou o pré-natal, sendo que 65,4% realizou entre seis ou mais consultas. Entretanto, observou-se que essa situagáo náo influenciou de maneira positiva para aumentar a autoeficácia em amamentar dessas mulheres. Apesar desses resultados, destaca-se que é justamente nas consultas de pré-natal que a gestante deve receber incentivo para amamentar seu bebe, sendo informada sobre os benefícios do leite materno e as desvantagens do desmame precoce^25^.

No que se refere ao recebimento de informagóes sobre aleitamento materno durante a realizagáo do pré-natal, é perceptível que mais da metade (50,7%) relataram náo ter recebido essas orientagóes durante as consultas, algo preocupante quanto a falta de informagóes prestadas durante a realizagáo do pré-natal.

O acompanhamento pré-natal é um momento primordial para estimular as máes a aderirem ao aleitamento materno, quando os profissionais precisam, além de ter competencias técnicas para desenvolver orientagóes a respeito da importancia, o manejo correto e as possíveis intercorrencias durante a amamentagáo, devem ter uma visáo ampla do contexto sociocultural, emocional e familiar da mulher ajudando-as a superar suas insegurangas/dificuldades e reconhecendo-a como a principal protagonista frente a lactagáo^25^.

Observou-se que apenas 25,0% das mulheres estudadas praticaram AME por seis meses na gestagáo anterior. A introdugáo precoce de alimentos náo é vantajosa para a saúde da crianga e quando isso acontece, ela pode apresentar episódios de diarreia, doengas respiratórias, além de correr o risco de ter desnutrigáo, visto que, há uma absorgáo prejudicada dos componentes do leite materno^25^.

O AME é considerado a melhor forma de alimentado para a crianza especialmente nos seis primeiros meses de vida e a prática é essencial para a diminuido das taxas de morbimortalidade infantil, e para a manutengáo das condigóes de saúde desta populado, entretanto, apesar de seus beneficios indiscutíveis para a saúde da crianga, ainda é pouco praticado^22^.

Com a aplicagáo da escala BSES-SF foi possível identificar que as máes apresentaram alta e média eficácia no pré e pós-teste, nenhuma delas obteve baixa autoeficácia para amamentar, sendo 64,0% puérperas com alta eficácia no pré-teste e 96,3% no pós-teste, indicando assim um aumento de 97 puérperas com alta eficácia depois da intervengáo. As análises estatísticas mostraram associagáo positiva (p-valor <0,001), mostrando que a atividade educativa com o álbum seriado representou uma tecnologia considerada positiva para aumentar a autoeficácia das mulheres em amamentar. Estudo que aplicou a mesma escala entre puérperas em hospital no centro-oeste do Rio Grande do Sul verificou também a ausencia de máes com baixa eficácia^26^.

Na presente pesquisa esse dado de náo obter puérpera com baixa autoeficácia pode ser explicado pelo fato de o hospital ser credenciado a Iniciativa Hospital Amigo da Crianga que promove, apoia e divulga a importancia do aleitamento materno em diversas etapas.

Pesquisa realizada com 132 puérperas em uma maternidade de Fortaleza (CE) a partir da aplicagáo da BSES-SF, mostrou que a maioria das mulheres estudadas apresentaram elevada autoeficácia em amamentar^27^. Pesquisa semelhante também encontrou efeito positivo com o uso do álbum seriado, e observou-se que as máes que antes da intervengáo apresentaram média autoeficácia, após, passaram a ter maiores escores de autoeficácia^26^.

O uso de uma intervengáo educativa sobre a autoeficácia da máe em amamentar, pode ser positiva como tecnologia educativa, em especial no momento do puerpério, quando a lactante necessita mais confianga em sua capacidade de prover as necessidades nutricionais de seu filho, o que facilita sua adesáo ao AME. A autoeficácia é um importante conceito que demonstra a confianga da máe em amamentar, conceito esse que trará impactos positivos no comportamento materno e nos indicadores de saúde da crianga^9^.

Os escores da autoeficácia em amamentar tendem a aumentar com o uso de uma tecnologia educacional, e maiores escores de autoeficácia sáo capazes refletir decisivamente nas taxas do AME^28^.

Uma pesquisa realizada a partir de atividades de educagáo em saúde em relagáo ao aleitamento materno verificou que após as intervengóes educativas houve modificagáo dos conhecimentos das gestantes em relagáo ao AME, contribuindo na efetividade do aleitamento materno e na redugáo da mortalidade infantil^21^.

Desta forma, percebe-se quáo relevantes sáo os profissionais de saúde que utilizam a educagáo em saúde como estratégia de incentivo a amamentagáo, que visam apoiar a nutriz desde o puerpério imediato, no período de internagáo no alojamento conjunto bem como nas consultas de acompanhamento de puericultura, com o foco em incentivar a confianga da máe, esclarecendo as dúvidas que possam interferir na prática do aleitamento materno^27^.

Como limitagáo deste estudo aponta-se a impossibilidade de analisar o impacto da intervengáo educativa sobre a prática da amamentagáo a longo prazo. Entretanto, para a saúde pública, a temática abordada no presente estudo foi relevante, incentivando o AME, diminuindo assim as taxas de desmame precoce.

## Conclusao

Os resultados desse estudo demonstraram que o uso do álbum seriado “Eu posso amamentar o meu filho” como estratégia educativa pautada na autoeficácia apresentou resultados significantes e associagáo positiva, visto que após a intervengo educativa houve um aumento dos escores da escala de autoeficácia para amamentar no puerpério imediato.

As mulheres estavam em idade fértil, tinham de nove a 12 anos de estudo, com renda familiar entre um e dois salários mínimos, eram donas de casa, tinham dois filhos, realizaram o pré-natal e menos da metade recebeu orientado sobre aleitamento materno no pré-natal.

Verificou-se que intervengóes educativas pautadas na autoeficácia podem ser relevantes para o apoio ao aleitamento materno, visto que, após a intervengáo educativa com o álbum seriado "Eu posso amamentar o meu filho” houve um aumento significativo dos escores da escala. Destaca-se entáo, a importancia dos profissionais de saúde frente ao desenvolvimento de intervengóes educativas a fim de minimizar as possíveis dificuldades do processo de amamentagáo, de forma que essas mulheres se sintam mais seguras e autoconfiantes em relagáo a amamentagáo.

É oportuno considerar o contexto em que a mulher vive, suas condigóes sociodemográficas, fatores culturais e psicossociais, considerando que, o desmame precoce pode ir além dos aspectos restritos ao acesso a informagáo.
